# Ensuring thermal comfort in the cabin of an electric vehicle at extremely low atmospheric temperatures (down to -45°C)

**DOI:** 10.1371/journal.pone.0337212

**Published:** 2025-12-02

**Authors:** Alexey V. Shvetsov, Svetlana V. Shvetsova

**Affiliations:** 1 North- Eastern Federal University, Yakutsk, Russia; 2 Togliatti State University, Togliatti, Russia; 3 Moscow Polytechnic University, Moscow, Russia; Stellenbosch University, SOUTH AFRICA

## Abstract

Ensuring thermal comfort for the driver and passengers in vehicles, including electric vehicles, when traveling on intercity roads, in sparsely populated northern regions with low traffic, in winter, at extremely low atmospheric temperatures is a critical factor. In this paper, we propose a method of providing additional or alternative heat to the cabin of an electric car, at atmospheric temperatures up to −45°C. To implement this method we developed, a backup electric heater for the cabin of an electric vehicle, which can provide autonomous heat generation for heating people in the cabin, even if the EV itself is completely inoperable. The backup heater is designed to use lithium-iron-phosphate batteries in its structure, which are safer than lithium-ion batteries. An experiment was conducted, as a result of which data were obtained on the discharge rate of the backup heater batteries when operating in three different air temperature ranges (from −20°C to −25°C, from −30°C to −35°C and from −40°C to −45°C) and the temperatures level reached in different parts of the EV cabin. Next, we compared the developed method with other known solutions and analyzed possible applications the backup heater.

## 1. Introduction

Northern regions of the planet, including Norway, Finland, northern provinces of Canada, the state of Alaska, and northern regions of Russia, such as Yakutia, Chukotka, etc., are a promising market for electric vehicle manufacturers [1]. However, promotion of electric vehicles in the markets of the northern regions is currently constrained by a number of factors, including reduction of reliability and safety of EV operation in conditions of low atmospheric temperatures, including extremely low temperatures (down to −45°C) [2–5]. Such temperatures are common in these northern regions during the winter season, and winter in the northern regions lasts longer than in other regions of the world and often lasts 5–7 months a year [6,7].

The average temperature in the northern regions during the winter season is usually −20...-30 degrees Celsius. At such temperatures the electric vehicle elements experience special stresses that can lead to accelerated malfunctions [8]. The elements most sensitive to low temperatures are those made of rubber and plastic, the physical properties of which undergo significant changes at low temperatures: they lose their plasticity, become more brittle and unstable to loads. This results in an increase in the number of failures in the on-board electronics of the electric vehicle. Energy consumption of the electric car battery increases both for maintaining the necessary temperature in the battery itself and for heating the EV cab, which leads to accelerated battery discharge and reduction of the total drive range of the electric car. At the same time, the impact of low atmospheric temperatures on electric vehicle components can be further exacerbated by strong winds, which are not uncommon in these latitudes.

Currently, the percentage of EVs in the total number of cars sold in the northern regions differs significantly from the number of EVs sold in regions with temperate and warm climates (Table 1) [9]. Data relative to 2023 are compared using two northern regions and two regions with temperate and warm climates located in the Russian Federation.

**Table 1:** % EM in total sales of passenger cars based on different types of power unit in 2023.

Sales volumes (by region)	Vehicles with conventional propulsion (fossil fuel)	Cars with a hybrid propulsion system	EV
Republic of Sakha (Yakutia) – northern region	90%	9%	<1%
Krasnoyarsk Krai – northern region	87%	11%	<2%
Moscow Region – temperate climate region	79%	17%	>4%
Krasnodar Krai – warm climate region	81%	15%	>4%

According to a survey of individual car owners and professional drivers of cab services conducted in the northern regions, four main factors deterring the main potential users from buying and using electric cars were identified (Table 2).

**Table 2:** Factors deterring the main potential users from buying and using electric cars in the northern regions.

Factors of refusal to buy EV	Individual car owners	Professional drivers of cab services
Insufficient charging and service infrastructure	27%	31%
Reduced EV power reserve at low atmospheric temperatures	26%	29%
Frequent occurrence of EV malfunctions under the influence of low atmospheric temperatures	22%	16%
Safety of traveling by EV in intercity trips in winter season	13%	11%
Risk of EV battery fire	2	5

As is seen from the survey results (Table 2), two of the five factors relate to EV safety. At the same time, the factor of EV safety in intercity travel in the winter season is specific to the northern regions.

These regions have low territorial population densities, which means that settlements are often located dozens or hundreds of kilometers away from each other. Traffic on the roads between such settlements is very low, again due to the low population density and the small number of businesses in the region. Thus, it is common for only one car to pass along the road in several hours, and in bad weather traffic stops altogether. On such road sections, the emergency stop of an electric vehicle in winter (e.g., in the event of a road accident, electric vehicle breakdown or battery discharge), followed by cessation of heat generation by the regular cabin heater, can lead to frostbite and death of people in the EV cabin from the effects of low atmospheric temperatures. Such an outcome is possible if help for their evacuation does not arrive in time, which is quite likely due to the remoteness of the nearest settlements and sparse traffic. Often in such regions there are heavy snowfalls with blizzards, which may also force the driver to stop the electric car and wait for help, for example, rescue teams or car service. Walking to the nearest settlement is again made impossible by the low atmospheric temperature, at which a person without special protective gear will get critical frostbite after only a few kilometers of travel.

Nowadays, some experienced motorists, take portable autonomous heaters running on gasoline or diesel fuel before driving through such dangerous areas [8]. But the use of such heaters, in turn, also creates risks for safety, because during their operation they emit carbon monoxide, which can be toxic if the proper venting of exhaust gases into the atmosphere from the cabin is not organized [10]. Moreover, the fuel on which such heaters operate is a flammable substance, and handling it requires special care, otherwise a fire may occur. Often such a portable heater is switched on by drivers in addition to the regular heater of the electric vehicle cabin because the regular heaters do not provide sufficient heat generation, as they are often not designed to heat the EV cabin in conditions when atmospheric temperatures reach −20°С...-45°С.

Various aspects of the theory of ensuring thermal comfort for the driver and passengers in vehicles, including electric vehicles, were previously addressed in works [11–30], they made a significant contribution to the development of models of thermal comfort in a vehicle, but they did not address the problem of reserving a heat source for the cabins of electric vehicles in case it is not possible to use the standard heating system.

In this paper, we propose a method of providing additional or alternative heat to the cabin of an electric car, operated in the northern regions of the planet at extremely low atmospheric temperatures (up to −45°C), based on the use of a safe energy source – electricity, in conditions where the heat generated by the standard heater EV is insufficient, or the standard heater is not operational.

## 2. Materials and methods

### 2.1. Method of providing additional or alternative heat to the cabin of an electric car

**Fig 1:**
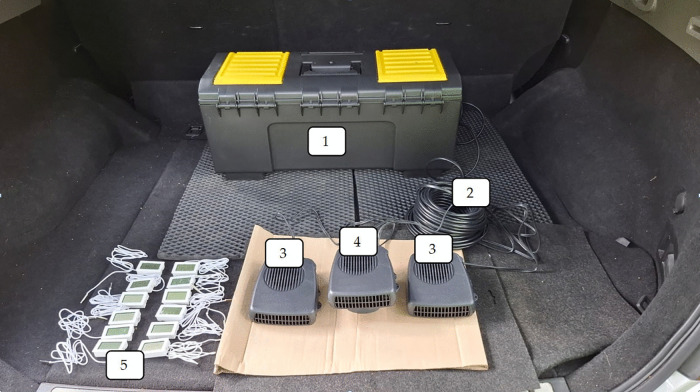
Equipment and materials: 1, 2, 3, 4 – elements of the backup heater, 5 – control equipment.

To solve the problem of providing additional or alternative heat to the cabin of an electric car, operated in the northern regions of the planet at extremely low atmospheric temperatures, we have developed a backup cabin heater powered by electric energy. The structure of the backup heater included an energy module containing 3 lithium-iron-phosphate batteries (LiFePO4) with a capacity of 100 Ah each. The total capacity of the module was 300 Ah. Lithium-iron-phosphate batteries were chosen for a number of reasons: non-flammability, durability (service life is > 2000 operating cycles), and frost resistance (batteries operate stably at temperatures to −45°C). Heat sources in the structure of the backup heater are 3 heat generators: 2 with a capacity of 200W and 1 with a capacity of 150W, operating from a 12V power supply. The complete structure of the developed electric vehicle cabin backup heater included 4 elements (Fig 1).

**Keys:**

1 – energy module (including three lithium-iron-phosphate batteries (LiFePO4);
2 – electric cable;
3 – heat generators (200Вт 12V);
4 – heat generator (150Вт 12V);
5 – temperature sensors.

**Fig 2:**
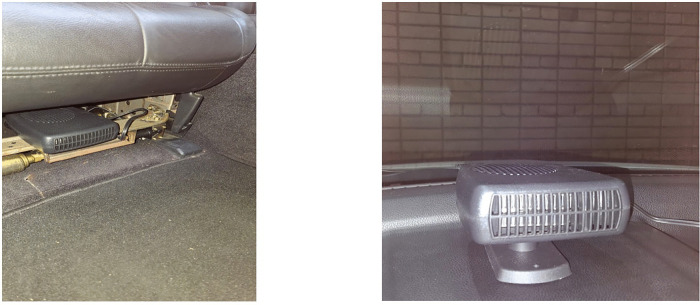
Locations for generators in the cabin. a) under the seat b) on the dashboard.

The principle of heat generation is as follows: air is heated by heat generators, which blow out heated air with the help of built-in fans. Locations for generators in the cabin were chosen based on the analysis of the coldest and most problematic zones, from the point of view of heating in the cabin of an electric car. One zone is the area around the driver and passengers feet (generators 1 and 2), and the second zone is the windshield area (generator 3) (Fig 2). The windshield is particularly susceptible to frosting on the inside due to the constant exposure of the windshield to external low atmospheric temperatures. Frosting-up on the inside of the windshield reduces the driver’s visibility significantly and often makes driving an electric vehicle impossible.

Starting the backup heater is carried out by the driver by pressing the starting toggle switches located on the heat generators.

### 2.2. Experiment

In to evaluate the backup heater, the method of field experiment was chosen. The field experiment should provide data on: a) the duration of the backup heater operation (based on data on the discharge rate of the backup heater batteries); b) the temperature level in the electric vehicle cabin achieved by heat generation by the backup heater.

The experimental use of the backup heater was carried out on two electric vehicles (Nissan Leaf brand), and at stage b) two similar electric vehicles were used as a control group, on which the temperature level was measured in the electric vehicle cabin achieved by heat generation by the regular heater.

The experiment was conducted at the test site in Yakutsk (region in North-East Russia), in winter, at three ranges of atmospheric temperatures:

a) −20°C…-25°С;
b) −30°C…-35°С;
c) −40°С…-45°С.

#### 2.2.1. Climate parameters during the experiment.

During the experiment, in addition to the basic measurements of atmospheric temperature, wind speed and air humidity were also measured to assess the general climatic conditions during the experiment (Table 3).

**Table 3:** The general climatic conditions during the experiment.

Atmospheric temperature (°C)	Wind (m/s)	Air humidity (%)
−20…-25	1-5	73
−30…-35	2-7	72
−40…-45	3-5	69

#### 2.2.2. Technical parameters of the experiment.

The experiment were carried out, we made three measurements of the backup heater operation duration at the same three ranges of atmospheric temperatures: −20°C...-25°C, −30°C...-35°C, −40°C...-45°C. Measurements were carried out simultaneously on electric cars No. 1 and No. 2. During the measurement, the electric vehicles were stationary with the regular heater switched off. Before switching on the backup heater, the air temperature in the cabs of electric vehicles was within the range of +10°C to −14°C. The “Timer” application on the Smartphone recorded the time from the moment the backup heater was turned on until the moment the heat generators stopped producing heat (as a result of the discharge of the backup heater batteries). Such measurements make it possible to obtain data on the duration of the backup heater operation at different atmospheric temperatures.

**Fig 3:**
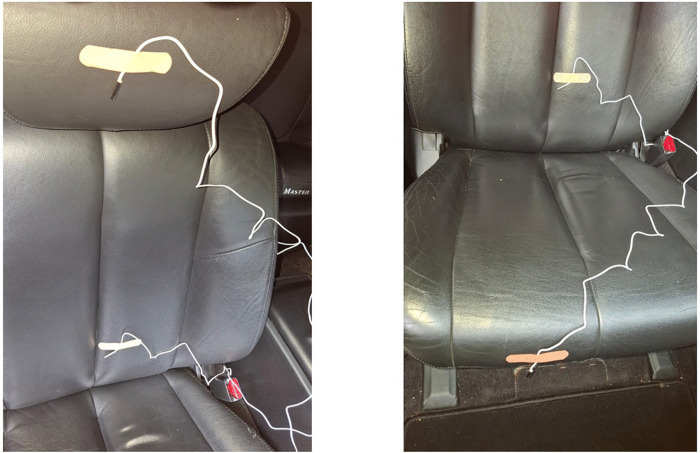
Placement of temperature sensors at the required points.

In parallel, the air temperature was measured in different points of the electric car cabin to assess the capability of the heater to maintain a sufficient temperature level. Measurements were made in 12 points: points 1–4 – the front part of the headrests of the driver and passengers; points 5–8 – the seat of the driver and passengers in the area of the human pelvis; points 9–12 – in the area of the shins of the driver and passengers, the location of points, clockwise, starting from the driver (Fig 3), temperature was measured after 30 minutes of uninterrupted operation of the backup heater.

**Fig 4:**
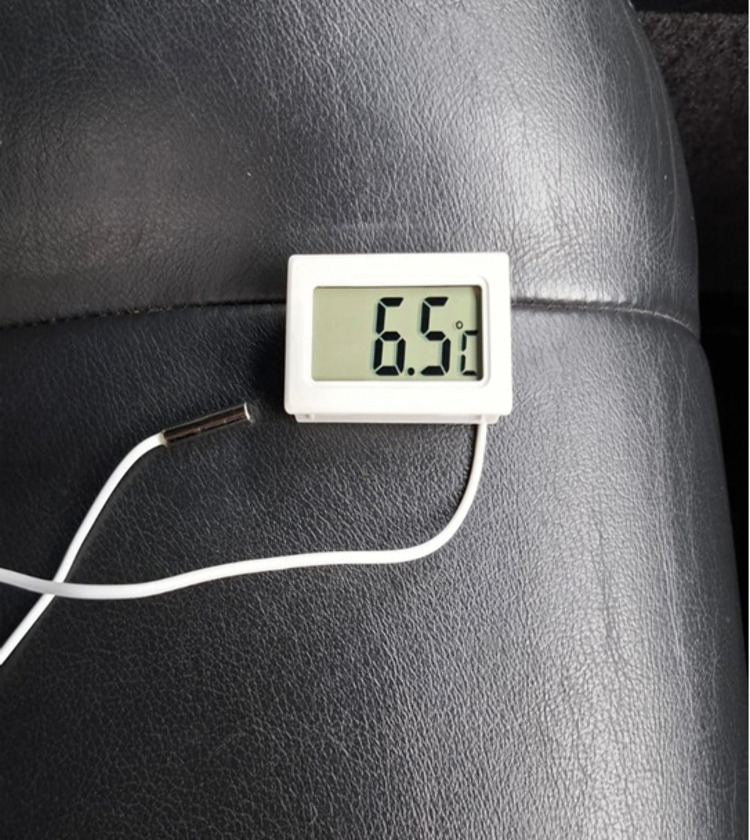
Temperature sensor digital display.

The temperature sensor readings were recorded visually based on the data displayed on the digital displays of the sensors (Fig 4). The accuracy of the temperature sensors used was 0.1 °C, temperature measurement range: from −50°C to +50°C.

## 3. Results

**Fig 5:**
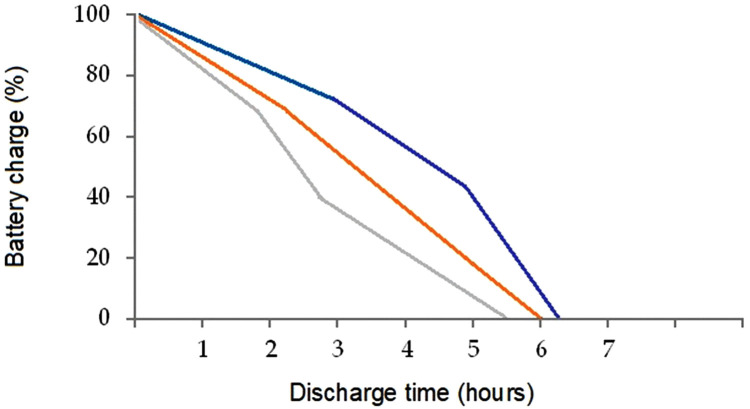
Discharge time of the backup heater batteries indicating the duration of the backup heater operation (the blue line on the graph is the discharge time at an atmospheric temperature of −20°C…-25°C, the orange line −30°C…-35°C, the grey line −40°C…-45°C).

The obtained median results for the duration of operation of the backup heater of the cabin are presented in Fig 5.

**Fig 6:**
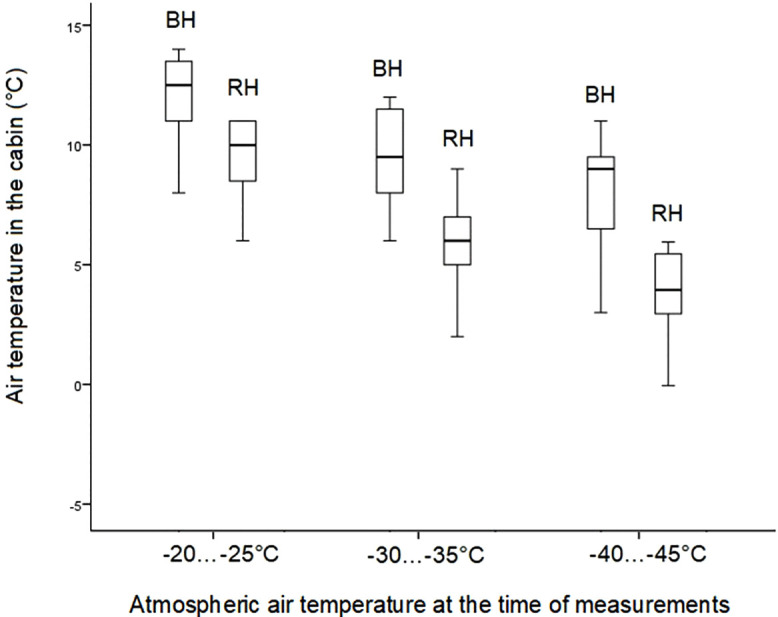
The results of measuring the temperature in the EV cabin with different heating methods (BH – temperature when the backup heater is operating, RH – temperature when the regular heater is operating).

The median operating time of the backup heater at various atmospheric temperatures is shown in Fig 6.

The result of the Mann-Whitney test:

A)  Analysis of measurement data at -20…-25°C:

$$U=min\{U_{\text{1}};\;\;U_{\text{2}}\}=\text{2}\text{5}$$

B)  Analysis of measurement data at -30…-35°C:

$$U=min\{U_{\text{1}};\;\;U_{\text{2}}\}=\text{1}\text{5}$$

C)  Analysis of measurement data at -40…-45°C:

$$U=min\{U_{\text{1}};\;\;U_{\text{2}}\}=\text{1}\text{5}.\text{5}$$

With *n*_*1*_ = 12, *n*_*2*_ = 12, *p* = 0.05, *U*_*kp*_ = 42.

The test results indicate that the temperature differences in the groups are statistically significant.

## 4. Discussion and conclusions

This paper proposes a method of providing additional or alternative heat to the cabin of an electric vehicle operated in the northern regions of the planet at extremely low atmospheric temperatures (down to −45°C). The method is based on generation of heat by a backup cabin heater. The heater allows the solution of one of the main problems of traffic safety in northern regions – it is the emergency stopping of an electric car on a road section, remote from populated areas, in conditions of low atmospheric temperatures, with the subsequent stoppage of the regular cabin heater, which threatens EV passengers with critical frostbite. As shown by our measurements, the temperature in the cabin of an electric vehicle after switching off the regular heater drops to critically low values (−0°C) in 18 minutes at atmospheric temperatures of −20°C...-25°C, in 12–13 minutes at −30°C...-35°C, and in 9–10 minutes at −40°C...-45°C. After that, the low air temperature leads to hypothermia of people in the EV cabin. The use of an emergency heater will provide the EV passengers with warmth while waiting for help. Under standard weather conditions, the time of arrival of help from a nearby settlement is 60–180 min, and it was experimentally established (Fig 5) that the operation time of the heater is 5.5–6.2 hours depending on the atmospheric air temperature.

The second task that can be solved by the developed backup heater is to maintain a comfortable temperature in the cabin of an electric vehicle when the heat generated by the regular cabin heater is insufficient. In most modern EVs, the heat generated by the regular heater is insufficient at atmospheric temperatures below −25°С. In such conditions, both regular and backup heaters can be used in parallel.

The third task solved by the backup heater is to increase the electric vehicle’s drive range, which is realized on the basis of saving the charge of the EV’s regular battery. This saving is achieved by using not the regular heater (which is a significant energy consumer of the electric vehicle’s regular battery) to heat the EV cabin, but by switching this function to the backup heater.

The backup heater is mobile and can be freely moved from one electric vehicle to another. For example, it can be used by different drivers on a “take-away” basis, where the driver takes the heater from a “Rescue Container” installed at the beginning of a dangerous section and returns the heater to a similar container at the end of the dangerous section of road. The rescue services fill the “Rescue container” with food, equipment, etc., i.e., with everything that drivers can take with them before driving along a dangerous road section and that will help them survive in case of a forced stop, including in difficult weather conditions, at extremely low atmospheric temperatures, until help arrives. Rescue containers are becoming increasingly common on road sections in northern regions, where distances between settlements are 30 km or more. Such road sections are subject to heavy snowfalls, blizzards and ice, which can force drivers to stop on the road. Often such roads are in forest or mountainous areas, which can also delay the arrival of rescuers to the stopped vehicles. There is very little car traffic on such sections, and this creates the risk that a broken-down car will not receive help from other drivers traveling on the same road. After passing a dangerous section of road, drivers return the equipment they have taken to the same “rescue container” located at the other end of the road section.

### 4.1. Applications

The backup heater can be used in the following practical applications (Table 4).

**Table 4:** Practical applications for the backup heater.

Application	Scenario
Assisting the standard heater in maintaining sufficient thermal comfort in the EV cabin	Under extremely low atmospheric temperatures, if the heat generated by the standard heater is insufficient to ensure thermal comfort in the EV cabin, the backup heater can be additionally (in parallel) activated as an auxiliary heat generation system.
Ensuring thermal comfort during long waiting periods for rescue operations	On intercity road sections in case of blizzards and snow drifts, during long waiting periods for rescue operations when the EV’s standard battery is discharged and the standard heater is inoperable, the backup heater can be activated to ensure thermal comfort in the EV cabin.
Increasing EV range	The standard EV cabin heater is turned off, which saves energy consumption from the EV’s standard battery and increases the EV’s range. Thermal comfort in the EV cabin during this period is provided by the operation of the backup heater. For sparsely populated northern regions, where distances between settlements can reach hundreds of kilometers, increasing the EV’s range can be a critical factor in choosing this vehicle.

### 4.2. Transformations for different vehicles and external conditions

The structure discussed is designed for a sedan-class EV, such as a Nissan Leaf, Tesla, etc. But the heater’s structure is dynamic and can be expanded for use in larger vehicle types, such as SUVs, minibuses, trucks, etc. The expansion conditions are related to the increase in the internal volume of the vehicle’s cabin, not its mass or overall dimensions. Thus, if a truck weighing 20 tons has a cabin volume no larger than a sedan’s, expanding the heater structure will not be required, as the heat will be sufficient for such a truck’s cabin.

In vehicles with a larger cabin, the heater structure can be transformed; for example, the number of batteries and heat generators can be increased to five, resulting in a 5x5 heater structure (five batteries and five heat generators). Changing the structure in terms of the number of batteries and heat generators used allows tailoring the backup heater’s configuration to any vehicle cabin volume, for example, structures can be 6x6, 8x8, etc.

The energy unit structure within the backup heater can also be changed to increase its effectiveness during extreme weather events, such as blizzards or road blockages, which may require long waiting periods for rescue operations. For such purposes, the heater structure can be modified as follows: the number of heat generators remains unchanged, while the number of LiFePO4 batteries in the energy module is increased, for example, in a 6x3 configuration (six batteries and three heat generators). This will increase the backup heater’s operating time to the required specifications, with the limitation being the increased dimensions and weight of the energy module. Such a transformation extends the backup heater’s operational capability for the anticipated waiting period for rescue operations, for example, from 5–7 hours (with a 3x3 configuration) to 10–14 hours (with a 6x3 configuration).

### 4.3. Placement and charging strategy

The use of a backup heater does not involve any modifications to the electric vehicle, which is important to maintain the validity of the manufacturer’s warranty and insurance.

**Fig 7:**
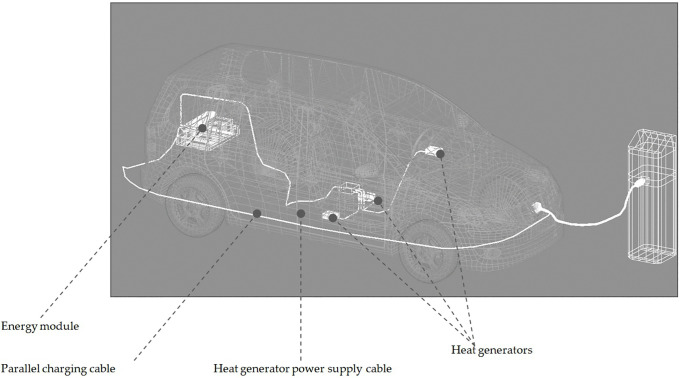
Location of the backup heater elements.

The following scheme for arranging backup heater elements in an EV is proposed (Fig 7).

### 4.4. Limitations

The backup heater energy module must be placed in conditions of positive atmospheric temperature, for example, inside the vehicle’s cabin or in a heated luggage compartment. This limitation is due to the fact that placing the energy module, for instance, in an open truck bed or an unheated cargo compartment, will lead to rapid cooling of the batteries after the EV leaves the garage and continues driving in extremely low atmospheric temperatures. Cooling the batteries to extremely low temperatures will reduce their discharge characteristics to critical levels, shortening the heater’s operating time by 50–90% (depending on the level of atmospheric temperature). The inability to place the heater’s energy module inside the vehicle’s cabin or in a heated luggage compartment may be due to the absence of a heated luggage compartment in certain vehicles or extremely limited free space inside the cabin.

It is important to note that the lithium iron phosphate (LiFePO4) batteries used in the backup heater experience significant performance degradation at low temperatures, characterized by a reduction in energy and power capabilities [31]. The main reason for the reduction in the discharge characteristics of LiFePO4 batteries is generally recognized to be lithium plating on the anode [31–34]. Lithium plating on the anodes causes interaction between the electrolyte and the deposited lithium (formation of lithium dendrites), which can lead to thermal runaway in the LiFePO4 battery and create safety threats, for example, causing a battery fire.

According to [31,35], a temperature drop to –30°C also leads to the solidification of the electrolyte and a sharp decrease in its ionic conductivity.

For this reason, the optimal location for the backup heater is inside the electric vehicle’s cabin, where a positive temperature is initially ensured both by the EV being in a heated garage (before the EV drives outside) and by the operation of the standard EV cabin heater, or the combined operation of the standard and backup cabin heaters. Adhering to this strategy ensures a positive battery temperature and preserves their discharge characteristics. If the EV is in extremely low temperatures and the standard battery is discharged, rendering the standard cabin heater inoperable, maintaining a positive temperature for the LiFePO4 batteries is ensured by the operation of the backup heater, which maintains a positive temperature in the EV cabin until the charge of the LiFePO4 batteries is depleted.

An important aspect is also how to isolate and place the LiFePO4 batteries in the vehicle. An important aspect is also how to isolate and place the LiFePO4 batteries in the vehicle. The problem of battery insulation in the proposed backup heater is solved by placing the batteries in the energy module, which is a larger plastic box. Energy transfer from the batteries to the heat generators is provided via electrical cables with double-layer electrical insulation.

The choice of location for the energy module containing the batteries is determined by the requirement that the placement location must ensure a positive temperature both when the EV is in the garage and when it is outdoors in extremely low temperatures. This can only be ensured by placing the Energy Module inside the EV cabin, with the luggage compartment being the optimal location.

The overall dimensions of the energy module (the main element of the backup heater) containing three plastic boxes with LiFePO4 batteries are 45x55x30 cm. These dimensions allow it to be placed in the luggage compartments of sedan-class electric vehicles.

### 4.5. Comparison with other known solutions

Currently, a number of methods for generating heat in an EV cabin are known. We have compared such methods and the backup heater proposed in this work (Table 5).

**Table 5:** Comparison of heating methods.

Method	Advantages	Disadvantages
Thermal Energy Storage Technology	Requires no additional costs for equipping the EV.	Reduced efficiency under extremely low atmospheric temperatures. Absence of this technology on most budget EV models. The technology will be inoperable in case of a complete EV breakdown.
Fuel Heater Technology	Possibility to replenish the energy source (liquid fuel).	Creates safety risks: – Carbon monoxide poisoning in case of improper exhaust venting from the vehicle cabin into the atmosphere; – Fire of the liquid fuel used in the heater, in case of improper handling or if the vehicle is involved in an accident.
Backup Heater	Use of a safe and environmentally friendly energy source – electricity.	The heater occupies part of the space in the EV cabin.

A promising direction for continuing this work is the development of more powerful batteries for the backup heater. Solving this problem will increase the operating time of the backup heater and the achievable level of thermal comfort in the EV cabin.
